# A Curious Case of Rectal Ejaculation

**DOI:** 10.7759/cureus.17330

**Published:** 2021-08-20

**Authors:** Frank L Ventura, Christopher M Nguyen, Alexander Dang, Michelle Baliss, Lindsay K Sonstein

**Affiliations:** 1 Internal Medicine, University of Texas Medical Branch at Galveston, Galveston, USA

**Keywords:** rectourethral fistula, foley catheter, urology, internal medicine, fistulas

## Abstract

Rectal-prostate fistulas are uncommon anatomical connections between the prostatic urethra and rectum that are typically iatrogenic but can also result from other underlying pathology. Here, we present a unique case of a rectal-prostate fistula causing the rectal passage of sperm.

A 33-year-old male with a history of illicit drug use presented with five days of testicular pain and a substantial amount of sperm passage from his rectum with ejaculation for the past two years. Computed tomography and voiding cystourethrogram (VCUG) of the pelvis revealed evidence of a rectal-prostate fistula. He was treated with piperacillin-tazobactam, and a surgical fistula repair was performed. Further investigation divulged a three-week comatose state due to cocaine and phencyclidine intoxication two years prior with documentation suggesting a traumatic Foley catheter placement and strong suspicion for premature balloon dilation in the prostatic urethra. Repeat VCUG revealed resolution of the fistula with mildly reduced antegrade ejaculatory volume.

Cases secondary to Foley catheter placement have not been previously reported in the literature. Even though urethral catheters have been shown to be effective tools in healthcare, it is crucial for clinicians to recognize the numerous potential complications that oftentimes become an afterthought to many providers. This case not only highlights a rare complication of catheter use but also emphasizes the importance of provider mindfulness when utilizing seemingly benign therapies such as Foley catheters.

## Introduction

Rectourethral fistulas (RUF) are uncommon pathologic communications between the rectum and lower urinary tract with an incidence of 0.5 per 100,000 per year [1]. The majority of adult cases are acquired, while most pediatric cases are due to congenital abnormalities [2-4]. Acquired RUFs occur due to surgery, radiation, trauma, and inflammatory states such as Crohn’s disease [2]. Most cases are related to prostate cancer or caused by prostate cancer therapy [5]. Presenting symptoms typically manifest as pneumaturia, fecaluria, hematuria, and urinary tract infections [5,6]. Management of these fistulous connections is complex and typically requires surgical correction with varying surgical techniques depending on the underlying etiology and surgeon preference [2,5]. Here, we describe a case of RUF causing the rectal passage of sperm due to traumatic Foley catheter insertion.

This article was previously presented as a meeting poster at the 2021 ACP National Abstract Competition in May 2021.

## Case presentation

**Figure 1:**
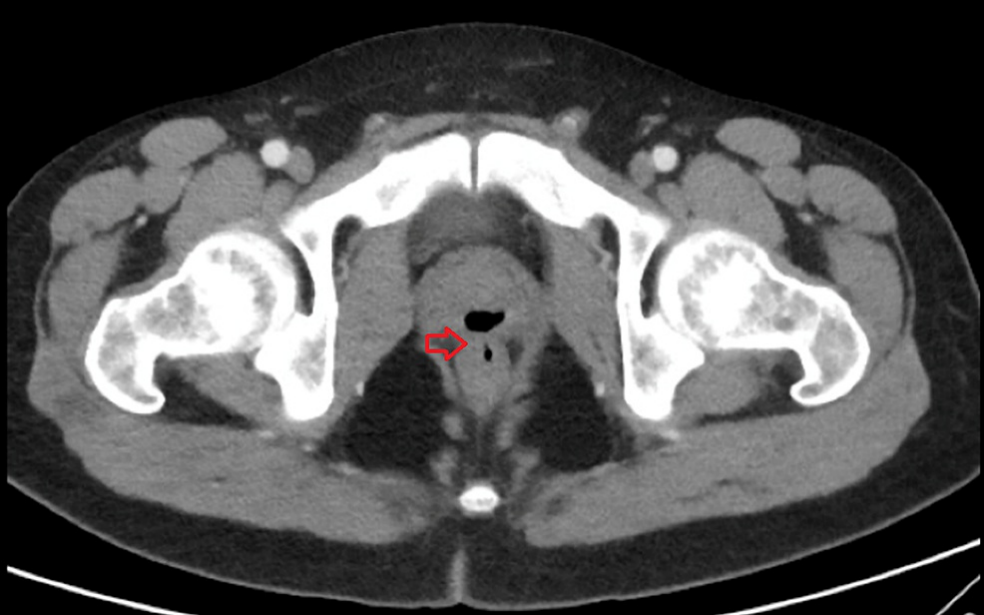
CT demonstrating gas-filled structure within the posterior aspect of the prostate communicating with the adjacent rectum. CT: computed tomography

**Figure 2:**
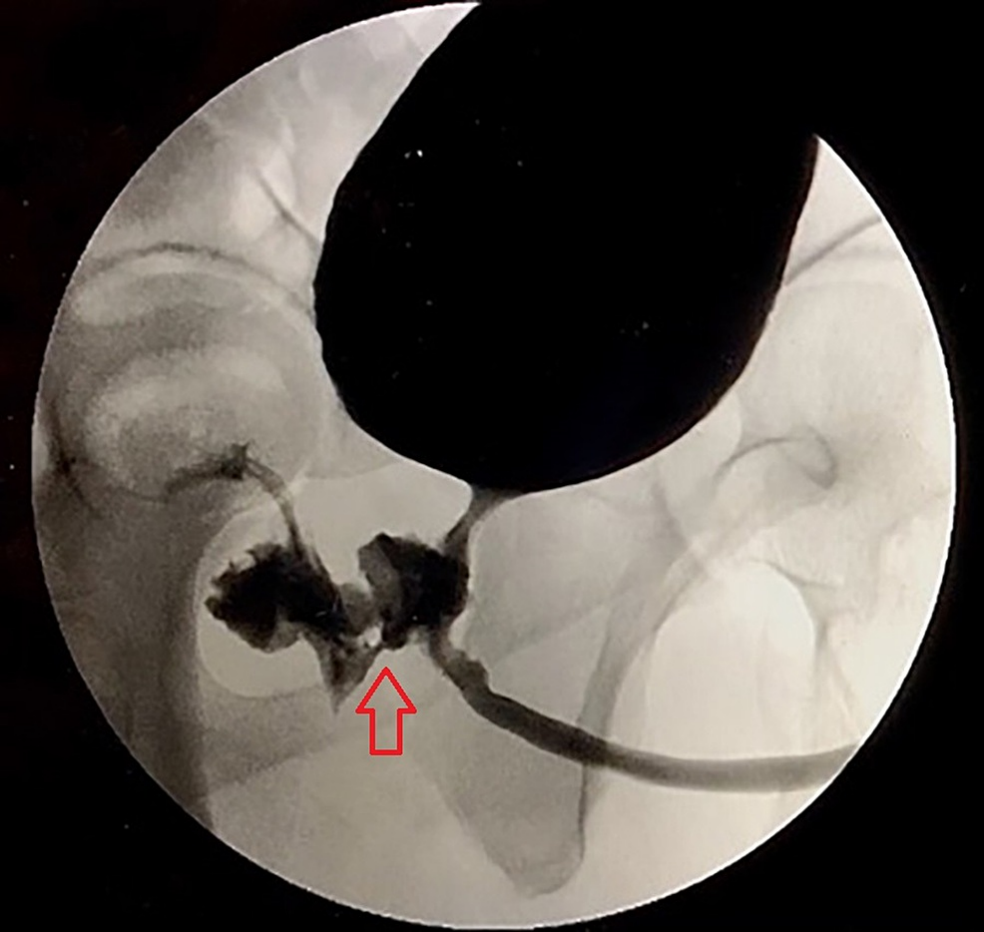
VCUG demonstrating evidence of fistulous communication of the urethra and rectum. VCUG: voiding cystourethrogram

A 33-year-old male with a history of illicit drug use presented with five days of testicular pain. He also noted a substantial amount of urine and sperm passage from his rectum in addition to pneumaturia and fecaluria for the past two years. Vital signs upon presentation were within normal limits. Physical examination was notable for left testicular swelling with a positive Prehn’s sign and a defect in the anterior rectal wall on digital rectal examination. Labs showed a neutrophilic leukocytosis and urinalysis was suggestive of a urinary tract infection. Computed tomography (CT) of the pelvis revealed evidence of left epididymo-orchitis and a gas-filled structure within the posterior aspect of the prostate communicating with the adjacent rectum, which was suggestive of a chronic appearing rectal-prostate fistula (Figure 1). Voiding cystourethrogram (VCUG) confirmed the presence of the fistula (Figure 2).

**Figure 3:**
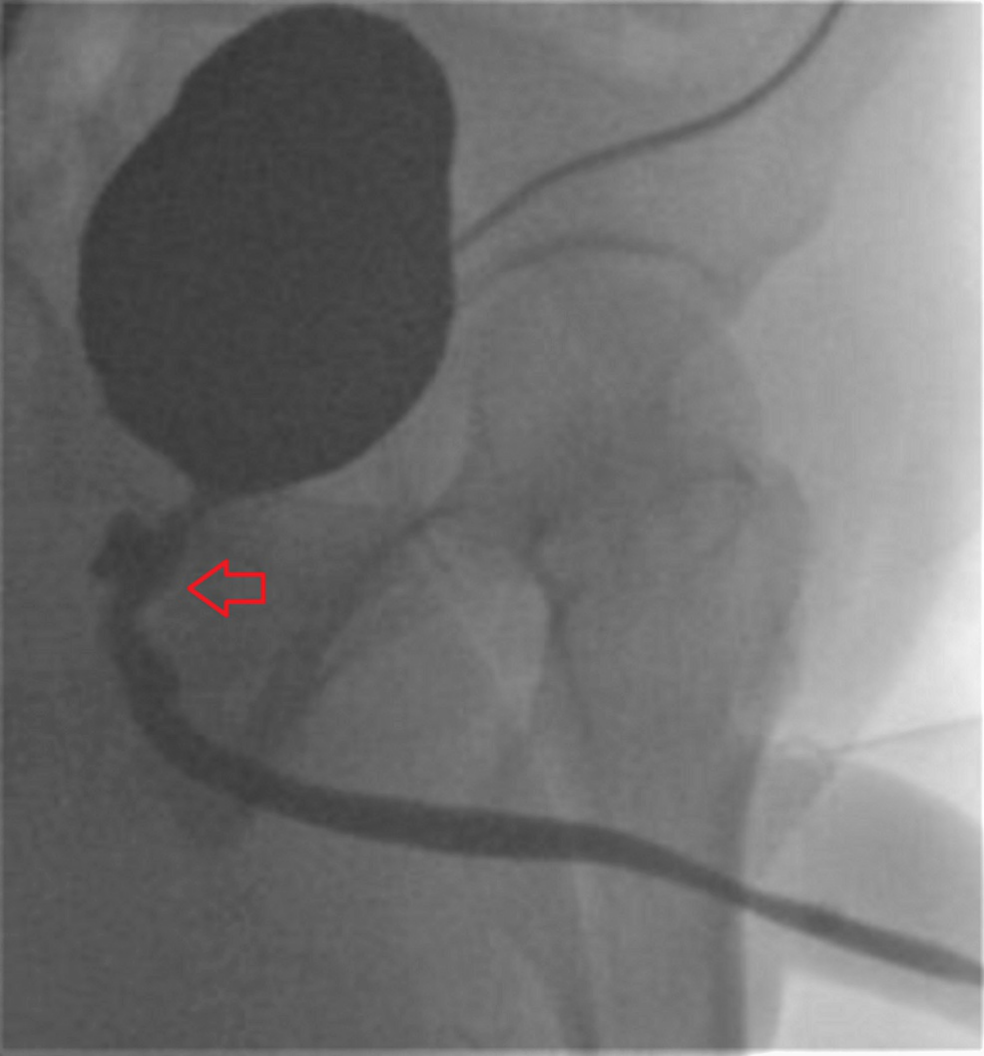
VCUG demonstrating evidence of fistulous resolution of the urethra and rectum. VCUG: voiding cystourethrogram

Extensive workup to rule out infectious causes including tuberculosis, inflammatory bowel disease, and malignancy was unremarkable. Additionally, the patient denied prior abdominal surgeries, rectal manipulation and penetration, or rectal trauma. Further investigation divulged a three-week comatose state due to cocaine and phencyclidine intoxication two years prior. Documentation suggested significant trauma with Foley catheter placement associated with hematuria during that hospitalization. The epididymo-orchitis was treated with piperacillin-tazobactam. A joint colorectal and urologic surgical fistula repair was performed creating a rectal urethral advancement flap from the excised rectal wall with successful closure of the prostatic fistula. A suprapubic catheter was placed postclosure and subsequently removed after recovery. Repeat VCUG revealed resolution of the fistula and the patient recovered with only mildly reduced antegrade ejaculatory volume over several months (Figure 3).

## Discussion

Several etiologies of acquired RUFs are well-established including prostate cancer with tumor invasion, rectal cancer, regional radiation, cryotherapy, surgery, diverticular disease, severe trauma, fistulizing Crohn’s disease, and abscess rupture (Table 1) [2,7,8]. Cases caused by trauma typically occur following severe trauma with a large number of cases occurring during times of war [9]. Catheter-induced RUF is rare with limited reported cases. Few cases of intraperitoneal urinary bladder fistulas, urethrocutaneous fistulas, and colovesical fistulas associated with long-term indwelling catheter use have been reported in the literature [10-12]. Untreated cases result in recurrent urinary tract infections, pyelonephritis, and sepsis [6].

**Table 1:** Cases of rectourethral fistulas with etiology. BT: brachytherapy; EBRT: external beam radiation therapy; FU: fecaluria; HC: hematochezia; HIFU: high-intensity focused ultrasound; MVA: manual vacuum aspiration; P: prostatitis; PU: pneumaturia; RP: radical prostatectomy; SPC: suprapubic catheter; TURP: transurethral resection of the prostate; UR: urine leakage through rectum

Investigators	Year	Number of patients	Age	Etiology	FU	UR	PU	HC	Surgery	SPC	Other
Koh et al. [13]	1997	1	73	TURP	-	+	-	-	+	-	-
Takaki et al. [14]	1997	1	60	PCa	-	+	-	-	+	-	-
Goto et al. [15]	2001	1	61	PCa	-	-	-	-	-	-	+
Kato et al. [16]	2006	1	65	RP	+	+	-	-	+	-	-
Kumar et al. [17]	2006	3	~52	P	-	+	-	+	-	+	-
Crippa et al. [18]	2007	8	~58	RP (5/8), debride (1/8) TURP (2/8)	+	-	+	-	+	-	-
Chiu et al. [19]	2008	1	70	EBRT	+	+	+	-	-	+	-
Khoder et al. [20]	2009	1	71	RP	-	-	-	-	-	-	+
Takezawa et al. [21]	2009	1	56	RP	+	-	-	-	-	-	+
Samalavicius et al. [22]	2012	1	58	BT	+	-	-	+	-	+	-
Xing et al. [23]	2016	1	75	P	-	-	-	-	-	-	+
Harris et al. [24]	2017	201	-	RP and ablation	+	-	-	-	+	-	-
Ubrig et al. [25]	2017	1	64	TURP	-	+	-	-	-	-	+
Moss et al. [26]	2018	1	80	BT	+		+	-		+	-
Prabha et al. [27]	2018	6	~54	MVA (2/6), RP (4/6)	+	+	-	-	+	-	-
Del Zingaro et al. [28]	2020	1	75	TURP	-	+	+	-	+	-	-
Kuperus et al. [29]	2020	1	70	EBRT	-	+	-	-	+	-	-
Pathak et al. [30]	2020	1	70	P	-	+	-	-	+	-	-

Symptomatic urinary tract infections are seen in 80% of patients with colovesical fistulas while approximately 90% of patients experience pneumaturia and fecaluria [31]. Rectal passage of sperm is a rare symptom with few established cases involving fistulous rectal connections with the ejaculatory duct or seminal vesicles due to surgical treatment of malignancy or inflammatory bowel disease [32-34]. The presence of feces in the urine is considered to be a poor prognostic sign as this may be an indicator that the fistula may be large [35].

A definitive gold-standard modality of diagnosis is not well established even though many cases are diagnosed by clinical suspicion followed by direct visualization by rectoscopy or cystourethroscopy [5,6]. Other imaging modalities such as CT, magnetic resonance imaging, and cystourethrography have also been used; however, subsequent direct visualization of the fistula typically occurs [6].

Conservative resolution by urinary or fecal diversion is variable ranging from 14% to 54% [5]. Due to the overall effect on the quality of life, and because most cases require surgical intervention for definitive therapy, conservative management is generally reserved for patients who are poor surgical candidates or who prefer a trial of less invasive management [5,34,36]. More complex cases may require surgical repair and postsurgical urinary or fecal diversion to facilitate optimal healing, as was the case in our patient [6,8]. Several known poor prognostic factors exist including fistulas larger than 2 cm and a history of regional radiation or cryotherapy [6]. Various successful surgical approaches and techniques for RUF repair have been established. A recent retrospective review found no significant difference in fistula healing following transanal, transperineal, transabdominal, or York-Mason repair with an overall 91% rate of successful repair [37]. Additionally, the study did not observe any difference in fistula healing in patients with or without urinary and/or fecal diversion [37]. Another study demonstrated multiple viable surgical approaches depending on the etiology, severity, and risk of recurrence [6].

This case not only highlights a rare complication of catheter use but also emphasizes the importance of provider mindfulness when utilizing seemingly benign therapies such as Foley catheters.

## Conclusions

The importance of Foley catheters in healthcare is well-established. While it serves many uses in patient management, it is essential to stay wary of its complications as well. More frequent complications such as recurrent urinary tract infections and kidney and bladder damage have been reported in the literature; however, physicians should note other potential risks such as urethral injuries and RUFs. This case not only highlights a rare complication of catheter use but also emphasizes the importance of provider mindfulness when utilizing seemingly benign therapies such as Foley catheters.
